# Understanding the Potential of Antibody‐Drug Conjugates Functionalized Engineered Exosomes in Hepatocellular Carcinoma Therapy: A Comprehensive Narrative Review

**DOI:** 10.1002/hsr2.73289

**Published:** 2026-09-25

**Authors:** Subham Sarkar, Avinaba Bose, Jenifer Rajak, Arup Kumar Mitra, Ajoy Kumer, Bikram Dhara

**Affiliations:** ^1^ Department of Biotechnology St. Xavier's College (Autonomous) Kolkata West Bengal India; ^2^ Department of Microbiology St. Xavier's College (Autonomous) Kolkata West Bengal India; ^3^ Department of Chemistry IUBAT‐International University of Business Agriculture & Technology Dhaka Bangladesh; ^4^ Department of Microbiology Saveetha Medical College and Hospital, Saveetha Institute of Medical and Technical Sciences Chennai Tamil Nadu India

**Keywords:** antibody‐drug conjugate, diagnosis, exosomes, hepatocellular carcinoma, targeted delivery, therapy

## Abstract

**Background:**

Exosomes are naturally secreted nanovesicles with high biocompatibility, low immunogenicity, and the ability to traverse biological barriers, making them attractive theragnostic carriers. When engineered to present tumor‐specific antibodies and to deliver antibody‐drug conjugates (ADCs), they may enhance selective targeting of hepatocellular carcinoma (HCC).

**Methods:**

This narrative review synthesizes the multidisciplinary field of exosome bioengineering, ADCs, and HCC diagnosis and therapy, covering topics from click chemistry to translational challenges. A comprehensive search of PubMed, Web of Science, and Scopus (1991–2026) identified relevant studies on exosome‐based targeted delivery, and diagnosis and therapeutics in HCC. Included studies focused on engineered exosomes, ADCs, and tumor‐specific targeting, with rigorous characterization and relevance to HCC diagnosis and therapy. The review aims to provide a conceptual synthesis, highlighting translational progress and future directions in engineered exosome‐based cancer diagnosis and therapy.

**Results:**

The exosomal lipid bilayer protects encapsulated therapeutic cargo from premature degradation in circulation, while antibody display confers high‐affinity recognition of HCC cells. A dual‐targeting paradigm leveraging both the exosome's inherent tropism and antibody specificity supports increased intra‐tumoral delivery and reduced off‐target exposure relative to conventional modalities. Co‐loading of imaging probes can enable integrated theragnostics, facilitating visualization of biodistribution, target engagement, and treatment response. However, as clinical data remains limited, these anticipated advantages in the human therapy should be established by thorough experimentation and clinical trials.

**Conclusion:**

ADC‐functionalized exosomes (immuno‐exosomes) can offer a suitable platform for precise HCC targeting with the potential to improve therapeutic index and enable real‐time response monitoring. Translational progress will depend on robust, scalable manufacturing, rigorous characterization of purity and potency, and comprehensive evaluation of safety, pharmacokinetics, and efficacy in preclinical models and clinical studies.

## 1 Introduction

Exosomes are a class of nanoscale extracellular vesicles, ranging from 30 to 150 nm in diameter, that are secreted from the endosomal system of nearly all cells [1, 2]. They act as essential mediators of intercellular communication, transporting a diverse cargo of biomolecules, including proteins, lipids, and nucleic acids (e.g., DNA, mRNA, miRNA, and lncRNA). This intrinsic ability to encapsulate and protect biological materials makes them a highly attractive platform for a new generation of cancer therapies, offering a more targeted and less invasive alternative to conventional treatments like chemotherapy and radiation [3]. The role of exosomes in cancer is a complex and fascinating double‐edged sword. While engineered exosomes hold immense therapeutic potential, their unmodified counterparts secreted by tumor cells can inadvertently promote cancer progression. These tumor‐derived exosomes can facilitate tumor growth by modulating the tumor microenvironment, inducing angiogenesis, and suppressing immune responses [4]. This dual nature underscores the critical importance of carefully engineering and modifying exosomes to harness their therapeutic power while neutralizing their pro‐tumorigenic effects. The emerging field of exosome‐based therapy is built on the principle of leveraging these nano‐vesicles' natural communication mechanisms [5]. By overcoming the limitations of conventional therapies, exosome‐based treatments are paving the way for more personalized and effective cancer care. One of the most compelling applications of exosomes is their use as precision‐guided delivery vehicles [6]. By modifying the exosomal surface, they can be directed to specific cancer cells, significantly reducing the systemic toxicity often associated with conventional chemotherapy drugs. For instance, expressing a peptide like iRGD on the exosomal surface allows them to specifically bind to integrins on the surface of tumor cells [7]. This targeted delivery ensures that a cytotoxic payload, such as doxorubicin or paclitaxel, is concentrated in the tumor microenvironment, maximizing its effect on cancer cells while sparing healthy tissue [8, 9]. Beyond carrying traditional drugs, exosomes can also be loaded with nucleic acids to perform advanced gene therapy. They can deliver siRNA, miRNA, or even CRISPR‐Cas9 reagents to specifically silence oncogenes or modify key signaling pathways, such as Wnt/β‐catenin and PI3K/Akt, that are crucial for cancer cell survival and proliferation [10, 11, 12, 13, 14]. This level of genetic precision offers a powerful new way to attack the core mechanisms driving cancer growth. Exosomes are also being utilized as nano‐vaccines to stimulate a potent and long‐lasting anti‐tumor immune response [15]. A particularly powerful approach involves using exosomes derived from dendritic cells (DCs), which are the immune system's most potent antigen‐presenting cells. These dendritic cell‐derived exosomes (DEXs) inherit the antigen‐presenting capabilities of their parent cells, carrying crucial surface molecules like MHC class I and II, co‐stimulatory proteins (CD80, CD86, and CD40), and tumor‐associated antigens (TAAs) [16]. When administered, these engineered DEXs can effectively educate the immune system by presenting tumor antigens to T lymphocytes, thereby activating a robust cytotoxic T‐lymphocyte response that specifically targets and eliminates cancer cells. This mechanism not only attacks existing tumors but also promotes immunological memory, which helps prevent disease recurrence [16]. Furthermore, exosome‐based nano‐vaccines are being investigated to counter epithelial‐mesenchymal transition (EMT), a process by which cancer cells become migratory and invasive [17]. By targeting antigens related to this process, these vaccines can potentially block a key pathway of metastasis, immune evasion, and treatment resistance.

## 2 Methods

This is a comprehensive narrative review. Given the rapidly evolving and multidisciplinary nature of exosome bioengineering, ADCs, and theragnostic in HCC, a narrative approach was selected over a systematic review. This allowed for a broader, conceptual synthesis of diverse topics ranging from click chemistry and surface engineering to preclinical translational challenges, which would be constrained by the narrow, single‐question focus required of a systematic review. To identify relevant literature, comprehensive searches were conducted across three primary electronic knowledgebases: PubMed, Web of Science, and Scopus. The search strategy employed combinations of relevant keywords and Medical Subject Headings (MeSH) linked by Boolean operators. The core search string included: (“exosomes” or “extracellular vesicles” or “nanovesicles”) and (“hepatocellular carcinoma” or “HCC” or “liver cancer”) and (“antibody‐drug conjugate” or “ADC” or “targeted delivery” or “surface engineering” or “click chemistry” or “diagnosis and therapy” or “imaging biomarkers”).

The literature search covered articles published between 1991 and 2026, capturing the most significant recent advancements in exosome isolation, nanomedicine, and targeted HCC therapies. Inclusion criteria consisted of: (a) peer‐reviewed original research articles and comprehensive reviews, (b) studies published in English, and (c) research explicitly investigating exosome tropism, ADC functionalization, tumor‐specific targeting, or theragnostic imaging in cancer models (with a primary focus on HCC). Non‐English publications, conference abstracts, editorials, or brief communications lacking full‐text data, studies focusing purely on unmodified exosomes without a theragnostic or targeted delivery context, and articles lacking rigorous characterization of the engineered nanocarriers, were excluded. The initial screening process involved evaluating the titles and abstracts of retrieved articles to eliminate duplicates and clearly irrelevant studies. Following this, the full texts of the remaining articles were independently reviewed for relevance to the core theme of the manuscript. Articles were ultimately selected for inclusion based on their scientific quality, conceptual relevance, and off‐target reduction, and their contribution to discussing the translational progress of engineered exosomes in cancer (encompassing HCC) diagnosis and therapy.

## 3 Exosomes as Theragnostic Agents in Hepatocellular Carcinoma

Hepatocellular carcinoma (HCC) is the most common form of primary liver cancer and is a major global health concern. Its prevalence is increasing, particularly due to the rising incidence of risk factors such as chronic hepatitis B and C infections, non‐alcoholic fatty liver disease (NAFLD), and liver cirrhosis [18]. HCC is notoriously difficult to treat for several reasons, which directly indicate the need for new cutting‐edge therapeutic approaches like exosome‐based therapies. A significant percentage of HCC cases are diagnosed at an advanced stage [19]. The early symptoms of liver cancer are often vague or non‐existent, and the disease typically develops in patients with pre‐existing liver conditions (like cirrhosis), which can mask the signs of a new malignancy [20]. This late diagnosis severely limits the effectiveness of curative treatments like surgical resection or liver transplantation [21]. Even after successful surgical resection, HCC has a high recurrence rate, with estimates ranging from 50% to 70% within 5 years [22]. This is often due to the presence of micro‐metastases that are undetectable at the time of surgery or the development of new tumors in the cirrhotic liver [23]. HCC is highly resistant to many conventional chemotherapy agents [24]. This intrinsic resistance makes systemic chemotherapy largely ineffective for advanced‐stage disease, leading to a poor prognosis. The molecular mechanisms behind this resistance are complex, involving multi‐drug resistance proteins, drug efflux pumps, and alterations in apoptotic pathways [25, 26]. HCC is characterized by significant inter‐tumor and intra‐tumor heterogeneity [27]. This diversity means that a single treatment is unlikely to be effective against all cancer cells, which can contribute to treatment failure and recurrence.

### 3.1 Exosomes as Nanoplatform for Targeted Therapy

**FIGURE 1:**
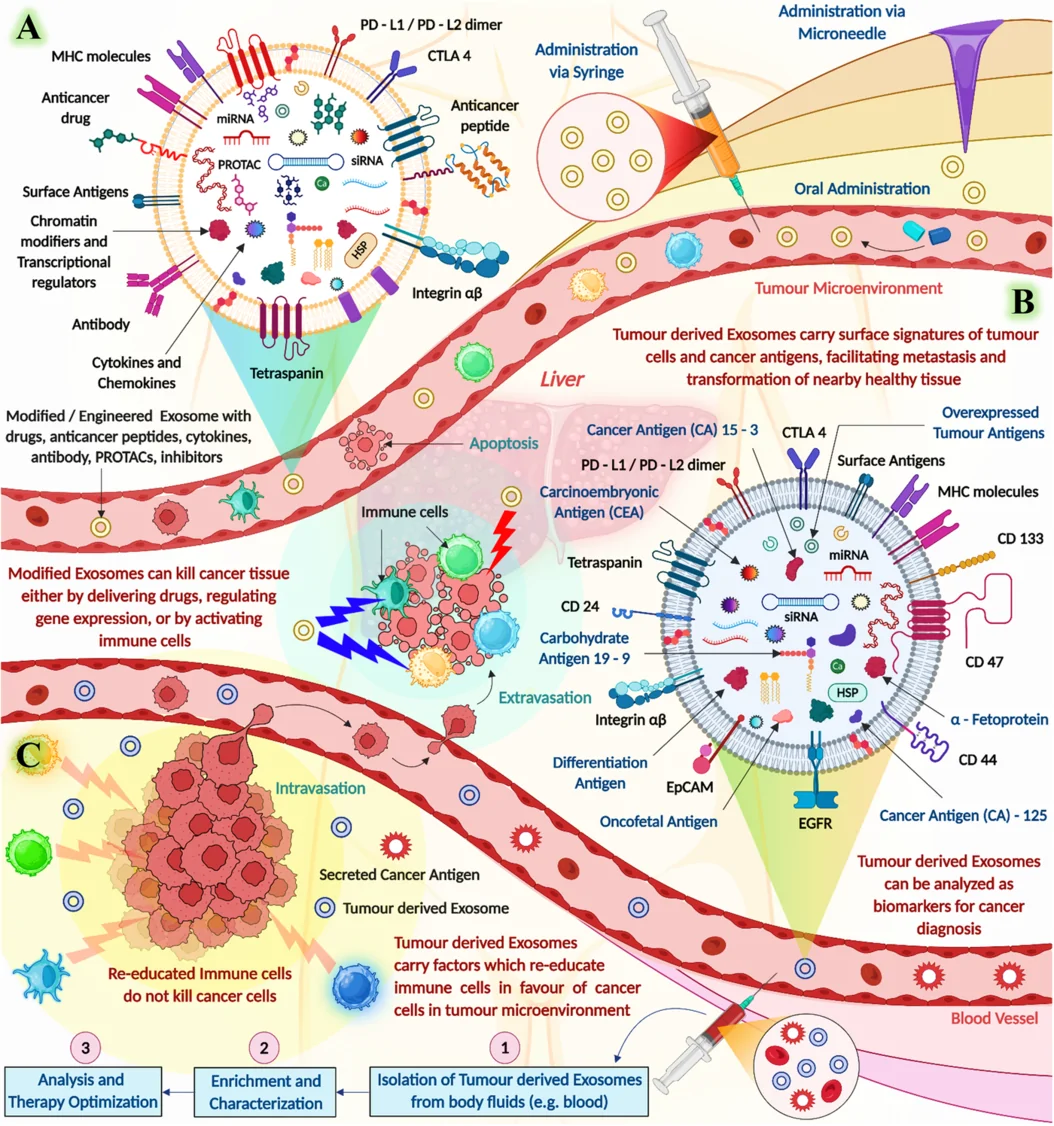
Exosomes as therapeutic and diagnostic nanoplatform for HCC. (A) Modified or engineered exosomes can be surface functionalized with antibody‐drug conjugates (drugs such as anticancer drugs, PROTACs, inhibitors which kill the tumor; antibody for targeted delivery). They can also contain anticancer drugs, PROTACs, and inhibitors in the lumen and release them when they get internalized by target tumor tissue. Modified exosomes (immuno‐exosomes as they have antibody‐drug conjugates) can kill cancer tissue by delivering drugs and inhibitors, blocking crucial surface receptors or ligands, or by re‐activating immune cells. Such exosomes have inherent tissue tropism and excellent biocompatibility. They can be administered orally, or intravenously, or via microneedles. (B) Tumor‐derived exosomes carry surface signatures of tumor cells (e.g., glypican 3, CD90, EpCAM, CD44, CD47, CD133, integrins, fucosylated Golgi protein 73, etc.) and cancer antigens (α‐fetoprotein, carbohydrate antigen 19‐9, oncofetal and differentiation antigens, etc.), facilitating metastasis, angiogenesis, transformation of nearby healthy tissue, and development of chemotherapy resistance. Tumor‐derived exosomes carry factors which reprogram immune cells in favor of cancer cells in tumor microenvironment (e.g., CTLA4, PD‐L1/PD‐L2, CXCR4, etc.). (C) Reprogrammed immune cells do not kill cancer cells and make tumor microenvironment immunosuppressive. Tumor‐derived exosomes can be analyzed as biomarkers for cancer diagnosis (processes include isolation of tumor‐derived exosomes from body fluids, enrichment and characterization, followed by analysis and optimization of therapy) (created in BioRender.com).

Given these challenges, exosomes are emerging as a promising and versatile tool in the fight against HCC. Their utility lies in their ability to serve as a multidimensional platform for both diagnostics and therapeutics [15]. Exosomes naturally exhibit some tropism for specific cell types [28]. Researchers can exploit or enhance this property to improve their targeting to the liver and HCC cells (Figure 1). One example is the modification of the exosome surface with specific peptides or antibodies that bind to receptors overexpressed on HCC cells. For instance, the iRGD peptide targets α_v_β_3_ integrin, which is often upregulated on the surface of HCC cells and tumor vascular endothelial cells [29, 30]. By decorating exosomes with iRGD, their therapeutic cargo can be guided directly to the tumor site, minimizing off‐target effects and systemic toxicity. Exosomes can be loaded with nucleic acids to specifically silence genes critical for HCC survival. For example, the Wnt/β‐catenin and PI3K/Akt signaling pathways are frequently hyperactivated in HCC, driving cell proliferation and survival [10, 13]. Exosomes carrying siRNA or miRNA that target key genes in these pathways can effectively inhibit their activity, leading to reduced tumor growth [31, 32]. This gene‐editing approach offers a level of specificity that is difficult to achieve with conventional drugs. The immune microenvironment of HCC is often immunosuppressive, which allows the tumor to evade detection and destruction by the immune system [33]. Exosomes can be used to reverse this phenomenon. DEXs, loaded with tumor antigens and co‐stimulatory molecules, can effectively prime the immune system, leading to a robust cytotoxic T‐cell response against HCC [34, 35]. Furthermore, exosomes can be engineered to carry immune checkpoint inhibitors (e.g., anti‐PD‐1 or anti‐CTLA‐4 antibodies) [36, 37]. By delivering these inhibitors directly to the tumor microenvironment, exosomes can reprogram T lymphocytes, allowing them to recognize and attack HCC cells that have previously been untargeted by the immune system. This targeted delivery of immunotherapies can enhance their effectiveness and reduce systemic side effects.

Microneedle patches offer a minimally invasive delivery route for modified exosomes, allowing for localized and sustained release into the skin's dermal and epidermal layers [18]. Direct administration via syringe, either intravenously or through intra‐tumoral injection, provides a high concentration dose of engineered exosomes to the systemic circulation or the tumor microenvironment, respectively [18]. While oral delivery presents significant challenges due to the inconducive gastrointestinal environment, current research explores strategies like exosomal encapsulation within enteric‐coated capsules to protect them and facilitate targeted absorption for systemic effects in HCC therapy [18].

### 3.2 Cancer Cell‐Derived Exosomes for Diagnosis of HCC

The cancer cell‐derived exosomes (CDEs) have important roles in promoting tumor growth and metastasis [38, 39, 40]. In HCC, these CDEs are not just passive byproducts, instead, they are active and sophisticated communicators that orchestrate the progression of the disease (Figure 1). CDEs carry a specific molecular cargo that reflects the genetic and phenotypic state of the parent HCC cell, effectively acting as messengers that reprogram recipient cells both locally and at distant sites. CDEs from HCC play a critical role in all stages of the disease, from initial tumor growth to metastatic spread, by influencing a variety of cell types within the tumor microenvironment (TME) and beyond [41]. HCC‐derived exosomes are central to shaping a TME that is conducive to tumor growth [42, 43]. They can be taken up by various non‐cancerous cells in the liver, including hepatic stellate cells, endothelial cells, and immune cells. The CDEs can reprogram these cells, for example, by transforming fibroblasts into cancer‐associated fibroblasts (CAFs), which then secrete growth factors and extracellular matrix components that support tumor proliferation and invasion [44, 45]. To sustain their rapid growth, tumors require a constant supply of oxygen and nutrients [46]. HCC cells release exosomes that contain pro‐angiogenic factors, such as VEGF (Vascular Endothelial Growth Factor), FGF (Fibroblast Growth Factor), and various lncRNAs [47, 48]. When these exosomes are absorbed by endothelial cells, they trigger the formation of new blood vessels, a process known as angiogenesis, which is essential for tumor expansion and metastasis. EMT is a crucial process in metastasis where cancer cells lose their epithelial characteristics and gain a more migratory, invasive mesenchymal phenotype [49]. HCC‐derived exosomes can induce EMT in neighboring tumor cells by transferring specific oncogenic miRNAs (e.g., miR‐21), proteins, and signaling molecules [50]. This exosome‐mediated communication is a key mechanism by which HCC cells become more motile and capable of invading surrounding tissue. One of the most prominent and dangerous attributes of CDEs is their ability to prepare distant organs for colonization by cancer cells, a process called pre‐metastatic niche formation [51]. HCC‐derived exosomes can travel through the bloodstream and reach other organs, such as the lungs or bone, where they are absorbed by resident cells (Zhao et al., 2021). Their cargo, which includes specific proteins and miRNAs, can alter the local cellular environment, making it more hospitable for future HCC cells that might arrive there. This organ‐specific seeding of a pre‐metastatic niche explains the preferential metastasis of HCC cells to predictable locations. CDEs actively suppress the anti‐tumor immune response. They can carry immunosuppressive molecules, such as PD‐L1 (Programmed Death‐Ligand 1), and deliver them to immune cells like T lymphocytes and natural killer (NK) cells [47, 52]. The transfer of these molecules effectively deactivates the immune cells, preventing them from recognizing and destroying the cancer cells. This exosome‐mediated immune evasion is a significant barrier to the success of immunotherapies. HCC cells can transfer drug resistance to other cancer cells via exosomes [53, 54]. This can happen through the delivery of efflux pump proteins or miRNAs that silence genes involved in drug‐induced apoptosis, a key mechanism of chemoresistance. This horizontal transfer of resistance is a major factor in the limited efficacy of many systemic HCC treatments.

Exosomes from HCC cells encapsulate a wide array of proteins, including those involved in cell adhesion, signal transduction, and metabolism. Specific proteins, such as GPC3 (Glypican‐3) and AFP (α‐fetoprotein), are well‐known tumor markers for HCC, and their presence on or within exosomes can serve as a sensitive indicator of disease [55, 56]. Other exosomal proteins like ADAM10 and CD147 are also being investigated as potential biomarkers [57, 58]. Exosomal miRNAs are a major focus of research. They can act as powerful regulators of gene expression. In HCC, specific miRNAs, such as miR‐21, miR‐122, and miR‐155, are often overexpressed in CDEs [59, 60, 61]. These miRNAs can be transferred to recipient cells to promote proliferation, invasion, and angiogenesis. Their distinct expression patterns make them excellent candidates for diagnostic and prognostic liquid biopsies [62]. LncRNAs, such as lncRNA‐H19 and lncRNA‐HOTAIR, are also found within HCC‐derived exosomes [63, 64]. Exosomes also carry fragments of mRNA and DNA, which can include oncogenic mutations (e.g., in the TP53 gene) or amplified genes [65, 66]. The presence of these genetic markers in circulating exosomes provides a snapshot of the genetic landscape of the tumor. The unique molecular cargo of HCC‐derived exosomes not only drives disease progression but also offers a rich source of potential biomarkers for diagnosis, prognosis, and treatment monitoring. This is the foundation of exosome‐based liquid biopsy. Beyond early detection, the cargo within tumor‐derived exosomes can provide valuable prognostic information [62]. Specific exosomal proteins (e.g., elevated levels of certain growth factors) or nucleic acids can be correlated with tumor aggressiveness, metastasis, and recurrence risk [15]. This allows for personalized treatment planning and helps clinicians predict how a patient might respond to a particular therapy.

## 4 Antibody‐Drug Conjugate Functionalized Immuno‐Exosomes for HCC Therapy

**FIGURE 2:**
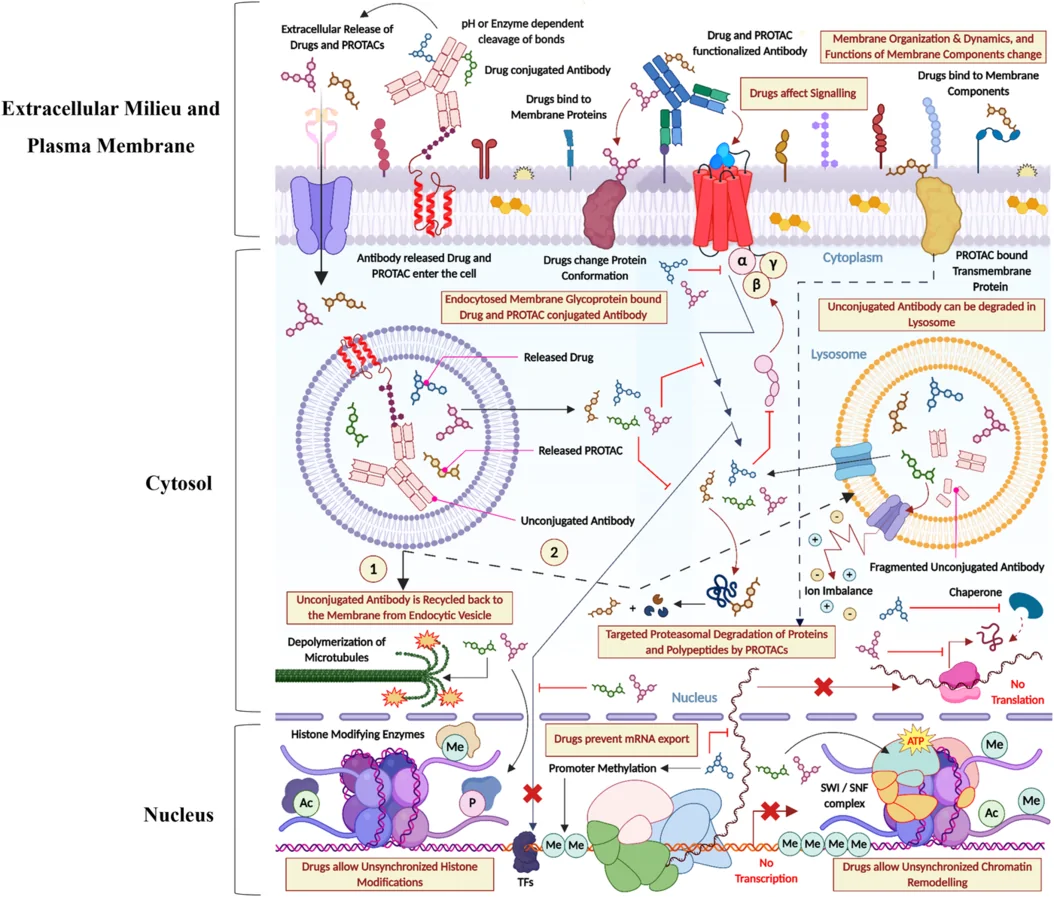
The administration of ADCs via immuno‐exosomes and immunoliposomes enables targeted delivery of drugs and PROTACs to cancer cells, leveraging the specificity of antibodies to navigate through biological barriers and accumulate in tumor tissues. The cumulative effect of drugs and PROTACs causes the impairment of indispensable cellular functions, ultimately leading to cancer cell death. In Extracellular milieu and at plasma membrane: these ADCs can be strategically positioned on the surface or encapsulated within the nanocarriers, allowing for controlled release of therapeutic payloads in response to environmental cues. The ADCs are conjugated to antibodies via cleavable linkers, which are designed to break down in response to changes in extracellular pH or upon internalization, releasing potent drugs or PROTACs. Once released extracellularly, these molecules can modulate signaling pathways, alter membrane dynamics and organization, change conformation of membrane proteins (ABC transporters), and block important moieties of crucial surface proteins and ligands (e.g., VEGFR), thereby disrupting cancer cell function. In Cytosol: upon internalization, the acidic environment of endocytic vesicles triggers linker cleavage, liberating drugs that depolymerize microtubules (e.g., Vinca alkaloids), cause protein misfolding by blocking chaperones (e.g., HSP90 and HSP70 inhibitors), and affect protein homeostasis, while PROTACs (e.g., ARV‐825 targeting BRD4 in HCC [67]) specifically target and degrade proteins essential for cancer cell survival. The resulting antibody which is no longer conjugated with drugs or PROTACs can either be fragmented and degraded with the vesicle, or can be recycled back to the cell membrane. In Nucleus: the liberated drugs translocate to the nucleus, disrupt chromatin remodeling and histone modification synchronization (e.g., HDAC inhibitors), block the recruitment of important transcription factors (e.g., BRD4 inhibitors), methylate promoters (e.g., Temozolomide), and prevent mRNA export and translation (e.g., XPO1 inhibitors preventing export, mTOR or eIF inhibitors preventing translation) (created in BioRender.com).

The success of immunoliposomes in targeted cancer therapy has established a powerful paradigm, that a biocompatible nanocarrier functionalized with an antibody to precisely deliver a cytotoxic payload can be used as immunoliposome analogs for antibody‐drug conjugate (ADC) delivery (Figure 2). Immunoliposomes are essentially synthetic lipid vesicles that have been surface‐decorated with antibodies to enable active targeting [68]. The process involves encapsulating a drug within the liposome's aqueous core and attaching antibodies to its lipid bilayer. This design has proven effective in delivering drugs to specific cancer cells, minimizing systemic exposure [68]. The synthetic nature of immunoliposomes generally triggers the accelerated blood clearance (ABC) phenomenon upon repeated dosing and can result in off‐target accumulation in the mononuclear phagocyte system, particularly the healthy liver and spleen. Moreover, the rigid structure of liposomes often limits their tumor accumulation and penetration deep into dense fibrotic HCC microenvironments. In spite of these disadvantages, immunoliposomes are preferred because they have been optimized for decades, offering highly standardized, scalable manufacturing and high drug loading efficiencies. Through PEGylation, their circulation half‐life can be extended significantly, allowing for prolonged systemic circulation. Exosomes, as natural nanocarriers, offer advantages to the disadvantages of immunoliposomes. Unlike synthetic liposomes, exosomes are naturally derived vesicles from human cells. This means they are inherently more biocompatible, have a lower risk of eliciting an immune response, and are less likely to be cleared rapidly by the reticuloendothelial system (RES) [69, 70, 71]. For liver‐related diseases like HCC, this is particularly advantageous, as it can reduce non‐specific uptake by the liver's resident macrophages (Kupffer cells), thereby improving tumor‐specific delivery [72] and having high tumor penetration. Exosomes possess a native ability to communicate with specific cells and can cross biological barriers more effectively than many synthetic particles [73, 74]. This intrinsic tropism can be exploited to enhance delivery to liver tissue. By engineering exosomes from a specific parent cell type or modifying their surface to enhance their homing capabilities, scientists can achieve more efficient and concentrated delivery of ADCs to the HCC tumor microenvironment. However, the biological advantages of immuno‐exosomes over immunoliposomes are currently offset by the technical and translational challenges in scalability, optimization constraints, and regulatory readiness (Table 1).

**TABLE 1:** Differences between immunoliposomes and immuno‐exosomes. Although immuno‐exosomes have regulatory and translational challenges, and lower drug loading efficiency compared to immunoliposomes, their low immunogenicity, high biocompatibility, and high tumor penetration capability make them advantageous for targeted drug delivery. Further investigations can establish immuno‐exosomes as suitable drug nanocarriers with optimized and convenient scalable manufacturing techniques.

Feature	Immunoliposomes	Immuno‐exosomes
Drug loading efficiency	High, easily controllable via remote loading.	Low to moderate, limited by endogenous cargo and lumen space.
Half‐life	Long (more than 30 h via PEGylation), but subject to ABC on repeated doses [75, 76, 77].	Short (minutes to hours) natively, but highly extendable by surface engineering (e.g., CD47 expression) without ABC [58, 78].
Tumor penetration	Limited, predominantly depends on the Enhanced Permeability and Retention (EPR) effect [79].	High, utilizes natural biological membrane fusion and intercellular transport mechanisms [80, 81].
Immunogenicity	Moderate to high; PEGylation (or other ligands used for functionalization) can induce antibodies [76, 82].	Low; homologous cell sources minimize immune rejection and complement activation [83].
Scalability & manufacturing	Highly scalable, established GMP protocols, low production cost [84].	Needs complex cell culture, difficult isolation (TFF/SEC), optimization constraints, requires stringent QC, high production cost [84, 85].
Batch‐to‐batch consistency	Precise control over lipid ratios and synthetic processes [86].	Variable; dependent on the metabolic state of producer cells [85].
Regulatory readiness	Well‐established FDA and EMA regulatory pathways for liposomal formulations [87, 88, 89].	As emerging complex hybrids, their regulatory pathways are still under development and currently lack the standardized frameworks associated with traditional liposomes.

High‐purity exosomes are first isolated from parental cells (e.g., genetically modified mesenchymal stem cells) using advanced techniques like size exclusion chromatography or immunoaffinity capture [15]. The ADC, which consists of an antibody linked to a cytotoxic drug or a PROTAC (Proteolysis Targeting Chimeras), is synthesized. The antibody's constant region is the ideal site for linking the drug or PROTAC via a cleavable linker, which remains stable in the bloodstream but is easily broken down by intracellular enzymes (e.g., lysosomal proteases) or stimulus (e.g., pH change in cancer microenvironment and endosome) [90, 91, 92, 93]. The ADC is then linked to the exosomal surface. This can be achieved through various methods, with click chemistry being a particularly promising approach. This bio‐orthogonal reaction allows for the precise and efficient conjugation of the ADC to the exosomal membrane without disrupting its structural integrity or functionality [37, 94]. This results in an immuno‐exosome that is functionally analogous to an immunoliposome but with the superior biological properties of an exosome. Once the ADC‐functionalized exosome is administered, it circulates until its antibody component recognizes and binds to a cognate receptor that is overexpressed on the surface of HCC cells. Following binding, the entire immuno‐exosome‐receptor complex is internalized by the cancer cell through receptor‐mediated endocytosis [95]. Once inside, the exosome is trafficked to the late endosomes and lysosomes. The acidic environment and high concentration of proteases in these organelles break the cleavable linker, releasing the free cytotoxic drug or PROTAC into the cell's cytoplasm [96, 97]. The released payload then initiates a specific mechanism of action to kill the cancer cell. The drug can inhibit cellular signaling, cause transcriptional and translational reprogramming, disorganize membrane fluidity, disrupt ion channels and transporters leading to ionic imbalance, and cause epigenetic modifications that inhibit cell growth and metabolism. If the payload is a PROTAC, it will selectively recruit the cell's own E3 ubiquitin ligase to the target cancer‐related protein, marking it for degradation by the proteasome [98]. This offers a highly effective way to eliminate key oncoproteins and prevent cell transformation (Figure 2).

## 5 Technical and Manufacturing Challenges of Exosome‐ADC Therapeutics

While engineered exosomes can offer profound advantages as theragnostic carriers for HCC, their clinical translation is currently delayed by several important technical and regulatory bottlenecks. A primary challenge lies in drug loading efficiency. Unlike synthetic lipid nanoparticles, exosomes possess an endogenous cargo of proteins and nucleic acids, leaving limited lumen space. Consequently, passive and active loading efficiencies for ADCs into or onto exosomes are significantly lower than the high loading efficiencies often achieved with synthetic liposomes. Furthermore, ensuring stability after chemical conjugation is difficult. Surface engineering and functionalization techniques, such as click chemistry, risk altering the exosome membrane integrity, potentially leading to vesicle aggregation, premature drug leakage, or the masking of native homing ligands essential for liver or, in general, organ tropism.

Manufacturing these complex biological therapeutics in a large scale presents another massive challenge. Scalability and GMP (good manufacturing practices) ‐compliant manufacturing are currently slowed down by the lack of standardized, high‐yield isolation protocols. While ultracentrifugation remains a gold standard in preclinical settings, it is unsuitable for large‐scale production. Transitioning to scalable methods like tangential flow filtration coupled with size exclusion chromatography is necessary but requires rigorous optimization to preserve vesicle integrity. Scientists have developed nanotechnology‐enhanced advanced microfluidics, but their optimization and further sophistication are needed to isolate and enrich specific populations of exosomes in industrial settings. This directly links into the issue of batch‐to‐batch variability and quality control. Because exosomes are cell‐derived, their composition changes based on the metabolic state of the cells from which they originate. Establishing robust quality control metrics that align with the Minimal Information for Studies of Extracellular Vesicles (MISEV) guidelines is necessary to guarantee consistent therapeutic efficacy and safety. The regulatory landscape for exosome‐ADCs is also vague and ambiguous. Because they combine a biological nanocarrier (exosomes), a targeting moiety (antibodies), and a cytotoxic payload (anticancer drugs), they do not fit ideally into existing regulatory frameworks established by the FDA or EMA. Defining clear criteria for their pharmacokinetic profiling, toxicity, and definitive mechanisms of action remains a significant regulatory challenge that must be addressed before widespread clinical testing can begin.

## 6 Conclusion

The concept of using engineered exosomes for ADC delivery represents powerful extension of the immunoliposome paradigm. It leverages the best features of both systems: the precision targeting of ADCs and the superior biocompatibility and delivery capabilities of exosomes. For HCC, this approach holds immense promise for overcoming current therapeutic limitations. The physiological complexities of the human tumor microenvironment, human‐specific systemic clearance mechanisms, and long‐term immunogenic responses cannot be fully recapitulated in current preclinical models. Consequently, significant challenges remain, including the need for scalable and reproducible methods for exosome isolation, the development of stable and efficient conjugation methods, and a thorough understanding of the biodistribution and ultimate fate of these engineered exosomes in the human body. Therefore, it is necessary to establish a clear evidence boundary regarding the current state of the field. The assertion that immuno‐exosomes (functionalized with ADC) definitively improve the clinical therapy of HCC patients remains speculative at this stage. While early translational data is highly encouraging, the clinical superiority of exosome‐based ADC delivery over traditional drug delivery platforms must be treated as a working hypothesis rather than an established clinical reality. Future breakthroughs will depend heavily on transitioning from bench‐scale proof‐of‐concept studies to rigorous, standardized, and GMP‐compliant clinical trials.

## Author Contributions

**Subham Sarkar:** investigation, conceptualization, writing – original draft, methodology, validation, writing – review and editing, software, formal analysis. **Avinaba Bose:** conceptualization, investigation, writing – original draft, data curation, software, formal analysis, methodology, validation, writing – review and editing. **Jenifer Rajak:** investigation, writing – original draft, writing – review and editing, software, formal analysis, data curation. **Arup Kumar Mitra:** conceptualization, formal analysis, project administration, supervision, resources, writing – review and editing, validation, visualization, investigation, funding acquisition. **Ajoy Kumer:** conceptualization, project administration, formal analysis, supervision, resources, writing – review and editing, funding acquisition, validation. **Bikram Dhara:** investigation, funding acquisition, conceptualization, writing – review and editing, visualization, methodology, validation, software, project administration, supervision, resources.

## Funding

The authors have nothing to report.

## Ethics Statement

The authors have nothing to report.

## Conflicts of Interest

The authors declare no conflicts of interest.

## Policy on Using ChatGPT and Similar AI Tools

During the preparation of this work, the authors used Grammarly for the purpose of English language editing. The authors reviewed and edited the content and take full responsibility for the content of the publication. No generative AI tools were used to draft, analyze data, or generate content for this manuscript.

## Data Availability Statement

All the data used in this study are present in the manuscript. The sources of data used for the preparation of the manuscript has been mentioned in the references. No new data were generated for this manuscript.

## Transparency Statement

Prof. Ajoy Kumer and Prof. Bikram Dhara affirm that this manuscript is an honest, accurate, and transparent account of the study being reported; that no important aspects of the study have been omitted; and that any discrepancies from the study as planned have been explained.
